# Small cell carcinoma of the ovary hypercalcemic type (SCCOHT): About three case reports

**DOI:** 10.1016/j.gore.2025.101932

**Published:** 2025-08-26

**Authors:** J. Benichou, J. Varinot, R. Bossi-Croci, M. Bazot, D. Sitbon, M. Dahan, C. Ferrier, Y. Dabi, C. Touboul, J. Lotz, E. Darai

**Affiliations:** a Department of Obstetrics and Gynecology, Tenon University Hospital, Sorbonne University, 4, rue de la Chine, 75020 Paris, France; b Department of Pathology, Pitié-Salpétrière University Hospital, Sorbonne University, 47-83 Bd de l’Hôpital, 75013 Paris, France; c Department of Radiology, Tenon University Hospital, Sorbonne University, 4, rue de la Chine, 75020 Paris, France; d Private Hospital Le Vert Galant, 38 rue de Flandre, Tremblay en France 93290, France; e Department of Oncology, Tenon University Hospital, Sorbonne University, 4, rue de la Chine, 75020 Paris, France

**Keywords:** Ovarian cancer, SCCOHT, Imaging, Chemotherapy, Radiation therapy, Survival

## Abstract

**Background:**

Small cell carcinoma of the ovary, hypercalcemic type (SCCOHT), is an extremely rare and aggressive ovarian cancer affecting young women, typically associated with poor survival. We present three cases of SCCOHT and review the current literature regarding clinical features, diagnosis, management, and survival.

**Cases:**

Three patients aged 16, 29, and 39 years were treated for ovarian masses. None presented with hypercalcemia. In case 1, MRI findings mimicked a mucinous cystadenocarcinoma, while the other two cases appeared as benign ovarian tumors. Following initial surgery and histological diagnosis of SCCOHT, all patients received comprehensive radical surgery, multi-agent chemotherapy, radiotherapy, and autologous stem cell transplantation. Two patients remain recurrence-free at 5 and 20 years, while the third has a 10-month follow-up. Genetic testing was performed in two patients, revealing no constitutional mutations in SMARCA4 or related genes.

**Conclusions:**

These cases highlight the difficulty of establishing a preoperative diagnosis of SCCOHT. Unlike previous reports, our data suggest that intensive multimodal therapy can result in favourable outcomes for stage I SCCOHT. Further studies are needed to define optimal treatment strategies.

## Highlights

• Diagnostic Challenges: Preoperative diagnosis of SCCOHT is difficult, often mimicking mucinous cystadenocarcinoma on imaging or other kind of ovarian tumors. Hypercalcemia isn’t always present.
• Intensive Treatment Matters: Intensive multimodal therapy, including surgery, chemotherapy, radiotherapy, and stem cell transplantation, is crucial for better outcomes.
• Genetic Insights: SMARCA4 mutations are common in SCCOHT and offer potential therapeutic targets (CDK-4/6 inhibitors, EZH2 inhibitors, or PD-L1 inhibitors). Genetic testing can be considered, though its value for at-risk relatives is debated.
• Improved Survival: With intensive multimodal treatment, patients with stage I SCCOHT do not systematically have a poor prognosis, showing improved survival outcomes compared to historical data.

## 1 Introduction

First described by Robert E. Scully in 1979, small cell carcinoma of the ovary, hypercalcemic type (SCCOHT), is an extremely rare and highly aggressive tumor. This malignancy accounts for less than 0.01 % of ovarian cancers (Kommoss et al., 2018, Lu and Shi, 2019). SCCOHT primarily affects adolescents and young adults, with a median age at diagnosis of 24 years, though cases have been reported from infancy to the early 40 s (Small Cell Carcinoma, 2025, Wens et al., 2023).

The hallmark of SCCOHT is its association with mutations in the SMARCA4 gene, which encodes a key subunit of the SWI/SNF chromatin remodeling complex (Tischkowitz et al., 2020). Loss of SMARCA4 (BRG1) protein expression is considered diagnostic for this entity. Despite its name, hypercalcemia is present in only 60–62 % of cases, making it an inconsistent diagnostic feature (Lu and Shi, 2019).

SCCOHT is notable for its rapid progression and is often diagnosed at an advanced stage due to non-specific symptoms. Prognosis is generally poor with disease-free survival below 10 % for advanced cases (Qin, 2018). Given its rarity and the young age at diagnosis, increased awareness, early detection, and effective treatment strategies are crucial.

We report three cases of SCCOHT that demonstrate some discrepancies from the existing literature.

### 1.1 Case 1

**Fig. 1:**
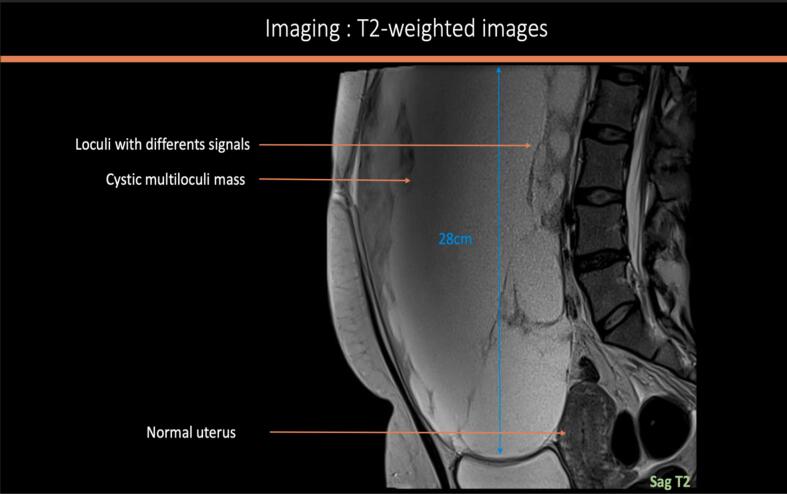

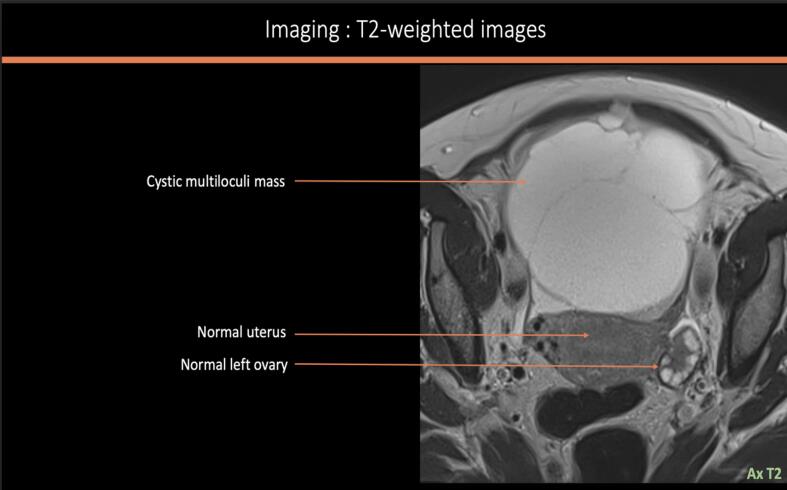

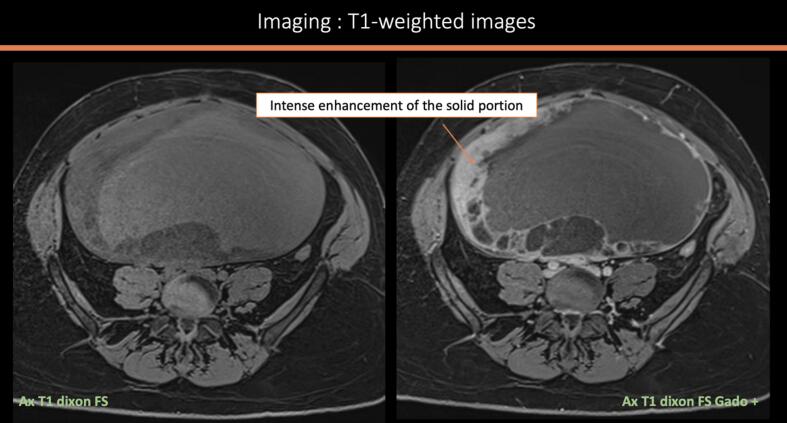

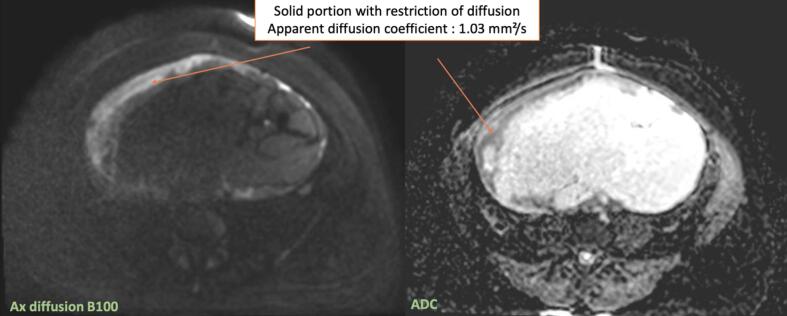
MRI factures of the tumor: Fig. 1a: sagittal section T2 weighted sequence showing a 28 cm cystic multiloculi mass with different signal Fig. 1b: axial section T2 weighted sequence showing a normal left ovary. Fig. 1c: Axial section T1 weighted image without (left) and with Gadolinium injection (right) detecting an intense enhancement of the solid portion Fig. 1d: Diffusion hypersignal (left) and diffusion restriction (right) of the solid component with diffusion restriction (ADC 1,03 mm^2^/s).

A 39-year-old woman with a history of three vaginal deliveries and one surgical abortion, and a family history of prostate cancer (paternal grandfather) and stomach cancer (paternal uncle), presented with a rapidly enlarging abdominopelvic mass and escalating pain over one month. Examination revealed a mobile abdomen mass measuring 40 × 30 cm with slight induration on the right flank. The vulva and cervix was normal, and the uterus was displaced by a large, fixed pelvic mass. Ultrasound revealed a multilocular abdominopelvic mass. MRI confirmed a large predominantly cystic right ovarian tumor measuring 28 cm, with locules of varying signals (intermediate T1–T2 signal, diffusion hypersignal, an ADC at 1.03 mm^2^/s, and homogeneous late contrast), thick irregular septa, and a solid component. The ORADS 5 appearance suggested mucinous cystadenocarcinoma (Fig. 1a–d). However, despite the absence of morphologically detectable signs of hormonal impregnation, the large peripheral tissue component should ruled out the possibility of tumors of the sex cords and secreting stroma (granulosa, Sertoli-Leydig tumor). CT scan confirmed a 29-cm right ovarian mass with hydronephrosis due to ureteral compression but no distant metastases. Serum CA 125 was 70UI/ml; others tumor markers including CA19.9 were negative. No hypercalcemia was detected preoperatively.

**Fig. 2:**
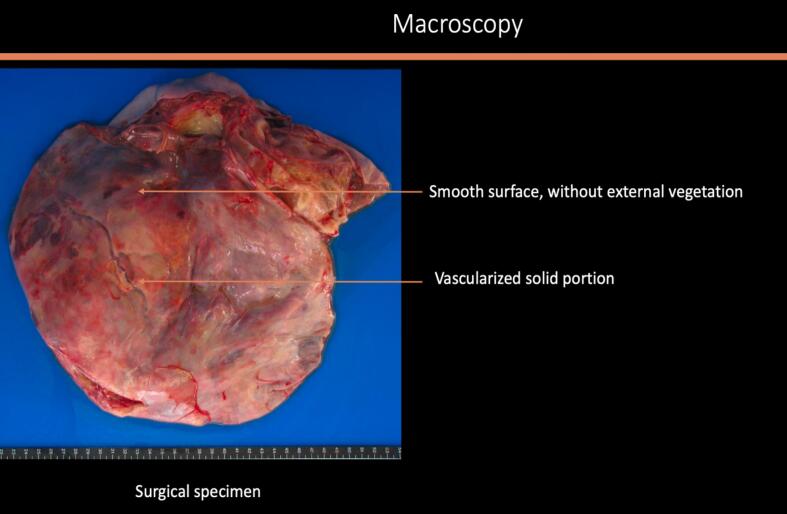

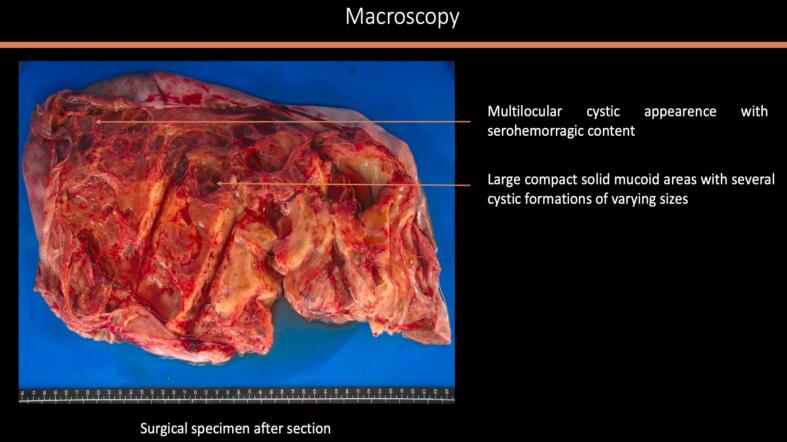

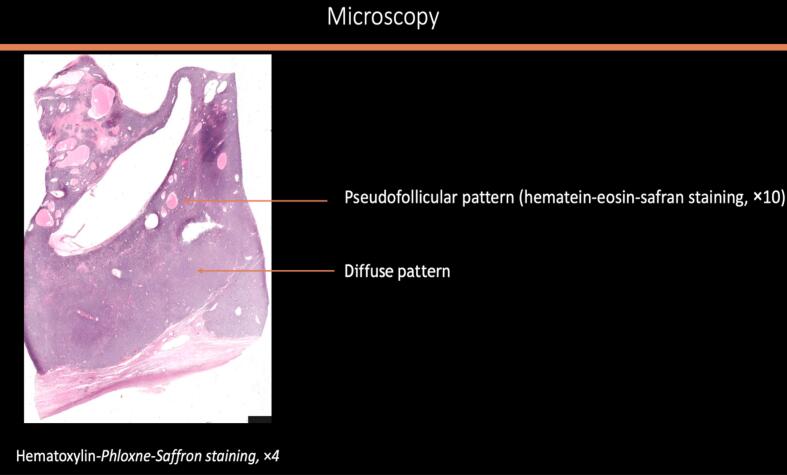

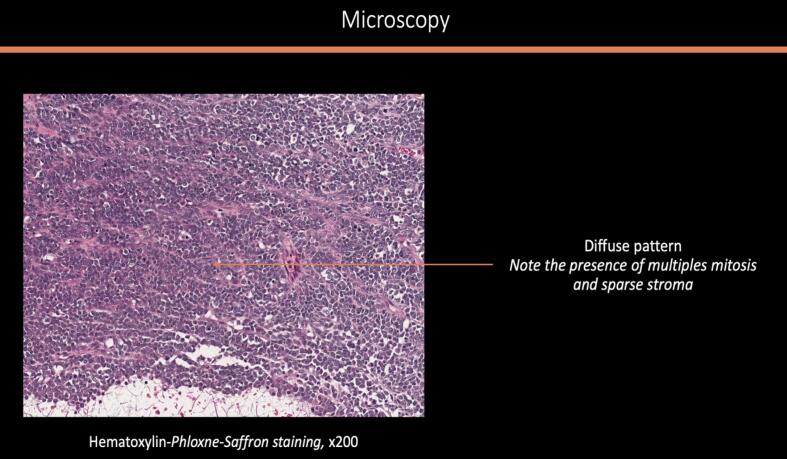

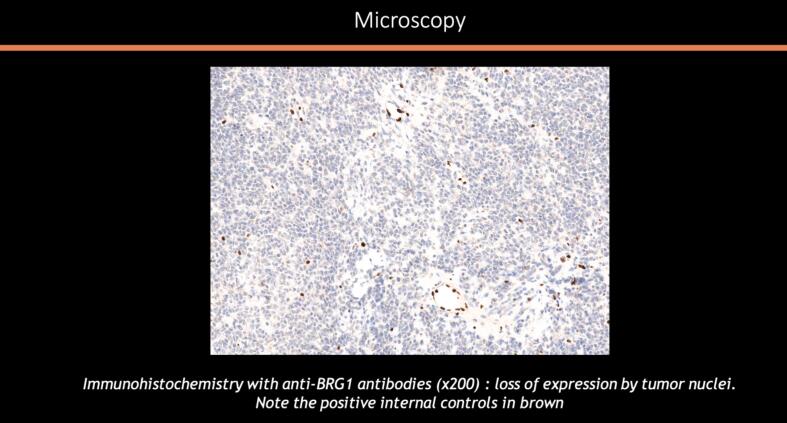
Histologic features of the tumor: Fig. 2a and b: macroscopic aspect of the surgical specimen showing multiple loci with thick septa. Fig. 2c and d: microscopic aspect of the tumor x4 (Fig. 2c) and × 200 (Fig. 2d) showing morphological appearance of small hyperchromatic cells with scant cytoplasm and brisk mitotic activity. Fig. 2e: Immunohistochemistry with anti-BRG1 antibodies (x200) exhibiting an intense and diffuse labelling.

A midline laparotomy was performed with peritoneal washing and right salpingo-oophorectomy. Intraoperative histology indicated an undifferentiated ovarian cancer, prompting immediate comprehensive cytoreduction (total hysterectomy, contralateral salpingo-oophorectomy, pelvic and *para*-aortic lymphadenectomy, appendectomy, peritoneal biopsies, and omentectomy). The tumor was staged as FIGO IA with no residual disease (R0). Histologically, the tumor was densely cellular with small to medium-sized cells, scant cytoplasm, and large hyperchromatic or vesicular nuclei. The mitotic index was high. In certain sectors, the cells appeared larger, with more abundant eosinophilic cytoplasm and large vesicular nuclei finely nucleolated. However, this substance was mucin negative. No endovascular tumor permeation was detected. On immunohistochemistry (IHC), the tumor was completely negative for all cytokeratins (pancytokeratin AE1-AE3, CK 5/6, CK 7, CK 10/13, CK 19), EMA, calretinin, hormone receptors, beta-HCG, alpha-fetoprotein, HMB 45, chromogranin A, inhibin, anti-desmin and panleukocyte antibodies. IHC showed complete loss of BRG1 (SMARCA4) expression, confirming SCCOHT. All other specimens were negative, confirming FIGO stage IA (Fig. 2a–e).

Adjuvant therapy included four cycles of PAVEP (cisplatin, doxorubicin, cyclophosphamide, and etoposide) followed by high-dose carboplatin, etoposide, and cyclophosphamide with stem cell autograft transplantation, and whole-abdomen radiotherapy (45 Gy).

Follow-up CT scans showed only a 31-mm lymphocyst, with no evidence of recurrence.

### 1.2 Case 2

A 29-year-old nulliparous women with no significant medical or family history, except for a prior laparoscopic ovarian cystectomy, presented with abdominal pain.

Ultrasound and MRI revealed a right ovarian mass measuring 10 × 10 × 12 cm. No ORADS classification or preoperative tumor markers were available. She underwent a right salpingo–oophorectomy abroad for a presumed FIGO IA ovarian cancer, without comprehensive staging. Initial histology suggested a stromal/sex cord tumor. Expert review (TENON AP-HP hospital in Paris and Lyon France) confirmed SCCOHT; SMARCA4 expression was not initially assessed. Postoperative CT and PET scans were normal.

After multidisciplinary oncologic committee evaluation and a delay of over six months since initial surgery (linked to the delay to obtain a visa), adjuvant chemotherapy was recommended followed by a cytoreductive surgery.

An initial PAVEP type 1 chemotherapy was performed (cisplatin one cycle 100 mg/m^2^ on Day 1, doxorubicine 40 mg/m^2^ on Day 1, etoposide 75 mg/m^2^ on Days 1–2–3, and cyclophosphamide 300 mg/m^2^ on Days 1–2–3). Three months later, a 72 × 73 mm necrotic *para*-aortic lymph node and peritoneal carcinomatosis was described on the CT scan. No thoracic metastasis was observed. Second line PAVEP type 2 (cisplatin four cycles 80 mg/m^2^ on Day 1, doxorubicine 40 mg/m^2^ on Day 1, etoposide 75 mg/m^2^ on Days 1–2–3 and cyclophosphamide 300 mg/m^2^ on Days 1–2–3) was given, resulting in complete response on CT and PET scans. Cytoreductive surgery was performed, including total hysterectomy, left adnexectomy, omentectomy, pelvic and lumbo-aortic lymphadenectomy and appendectomy. Histology confirmed the absence of residual disease. An intensification chemotherapy was done by epirubicin–paclitaxel protocol followed by stem cell autograft transplantation. No radiation therapy was performed, after discussion in a multidisciplinary committee.

Follow-up included clinical exams every 4–6 months, annual imaging, and CA125 testing. All results remained normal. Five years after the surgery, no recurrence was detected. Genetic testing revealed no constitutional mutation in SMARCA4 or SMARCB1.

### 1.3 Case 3

A 16-year-old patient with no personal or family history underwent a right salpingo–oophorectomy abroad for a presumed FIGO IA ovarian cancer without peritoneal washing or preoperative tumor markers. No data on imaging findings were available. Pathology review in our department (TENON AP-HP Paris), by pathologists of the Gustave Roussy Institut (Villejuif, France), and in the United States confirmed SCCOHT. Due to a 6-month delay before treatment in France, the multidisciplinary team recommended chemotherapy (etoposide and platinum-based agents), followed by comprehensive surgery (hysterectomy, contralateral salpingo–oophorectomy, omentectomy, peritoneal biopsies, and pelvic and *para*-aortic lymphadenectomy). Intensification chemotherapy and stem cell autograft, as well as radiotherapy, were administered postoperatively. Follow-up ultrasound over 9 years revealed no recurrence. Genetic testing 17 years after initial treatment showed no mutations in SMARCA4, BRCA1, BRCA2, or PALB2. She remains disease-free 20 years after diagnosis.

## 2 Discussion

Our case reports highlight the challenges of establishing a preoperative diagnosis of SCCOHT. Due to its rarity, the optimal adjuvant therapy—apart from surgery—remains debated. Notably, our findings suggest that the prognosis for SCCOHT is not always dire.

From a clinical viewpoint, in our cases, the main presenting feature was a rapidly growing abdominopelvic mass with pain, consistent with a recent review (Wens et al., 2023). None of our patients had a family history, although both germline and somatic SMARCA4 mutations have been reported in familial cases (Wens et al., 2023, Longy et al., 1996, Kupryjańczyk et al., 2013, Jelinic et al., 2014, Ramos et al., 2014, Witkowski et al., 2014). None of our patients had hypercalcemia, reinforcing that it is not required for diagnosis, as it is seen in only 36.4–60 % of cases (Callegaro-Filho et al., 2016). Matias-Guiu et al. found parathyroid hormone-related protein expression in four of seven SCCOHT cases (Matias-Guiu et al., 1994). Like other rare ovarian tumors (clear cell, endometrioid, Brenner, mucinous), SCCOHT is usually unilateral. Previous review indicate that SCCOHT often appears as a solid ovarian mass on imaging, mimicking dysgerminoma, granulosa cell tumor, or undifferentiated carcinoma (Blatnik et al., 2024). In contrast, our cases were predominantly cystic, resembling benign lesions or mucinous adenocarcinoma, with no specific features suggesting SCCOHT (Li et al., 2024). With the exception of tumors with hypercalcemia, these findings demonstrate the difficulty of preoperative diagnosis. All patients underwent follow-up with gynecological exams and imaging, though the benefit of this approach is debatable. In our small series, median follow-up was 84 months, including one recently treated case. Our results contrast with the review of Wens et al. reporting a five-year overall survival for patients with FIGO stage I of 51 % (95 % CI: 35–75), and 24 % (95 % CI:14–40) for patients with FIGO ≥ II (Wens et al., 2023).

From a surgical perspective, all cases were diagnosed at presumed FIGO stage IA, consistent with Wens et al., who found that 45.7 % of SCCOHT cases are diagnosed at stage I (Wens et al., 2023). International guidelines recommend radical surgery, adjuvant chemotherapy, and radiotherapy (Tischkowitz et al., 2020). However, the occurrence of SCCOHT in young women with unilateral tumors and no evidence of spread raises questions about conservative surgery There is no consensus on the need for pelvic and *para*-aortic lymphadenectomy. In our second case, incomplete staging was associated with early lymph node recurrence, highlighting the importance of thorough staging.

In addition to conventional histology, Mazibrada et al. reported seven SCCOHT cases with retained BRG1 (SMARCA4) expression by IHC, whereas previous studies indicate that loss of BRG1 is a key diagnostic criterion (Clarke et al., 2016, Karanian-Philippe et al., 2015, Mazibrada et al., 2023). Cyrta et al. recently showed that weak or faint staining is more indicative of splice site mutations, while BRG1-negative cases are associated with truncating mutations (Cyrta et al., 2024). Thus, weak or partial BRG1 loss on IHC remains consistent with SCCOHT. SMARCA4 is the main SWI/SNF complex member mutated in SCCOHT (Jelinic et al., 2014, Itamochi et al., 2017, Muppala et al., 2017), but other subunits (SMARCA2, SMARCB1, SMARCC1) may also be involved at low frequency (Itamochi et al., 2017, Simões et al., 2022, Ramos et al., 2014). Loss of SWI/SNF function can also result from post-translational silencing, as seen with SMARCA2 (Jelinic et al., 2016). This may explain the absence of SMARCA4 mutations in our cases. Gao et al. found that combined mutation and epigenetic silencing of SMARCA4 and SMARCA2 occur in the majority (69–100 % and 86–100 %, respectively) of SCCOHT cases (Gao et al., 2024). Wade et al., using UK Biobank data, found SMARCA4 germline pathogenic variants in 0.0038 % of females and 0.01 % of males, with higher prevalence in males (*p* = 0.028) and an estimated penetrance of 62 %. These findings provide important insights into the genetic basis and risk assessment of SCCOHT (Wade et al., 2025).

Concerning adjuvant therapy, no international consensus exists on the best option, or on the modalities of medical therapy and surveillance. A multimodal approach including radical cytoreductive surgery, platinum-based chemotherapy, whole-abdomen radiation therapy, and high-dose chemotherapy with autologous stem cell transplantation is often proposed as in our case reports (Young et al., 1994). However, it is important to note that various chemotherapy regimens have been used without the superiority of one regimen being shown (Table 1; 14, 25, 28–33). This is probably linked to the low incidence of SCCOHT and its classification as a miscellaneous neoplasm (RJ K, 2021) sharing features of rhabdoid tumors with SWI/SNF mutations associated with a poor response to conventional therapy (Brennan et al., 2013). Indeed, mutation and epigenetic silencing of SWI/SNF complex members are associated with the poorer prognosis and chemoresistance, especially in young patients (Brennan et al., 2013, Ma et al., 2024). Recently, other drugs have been suggested, based on pre-clinical studies with insufficient data to clarify the indications (Table 1; (Blatnik et al., 2024, Gao et al., 2024, Wang et al., 2017, Jelinic et al., 2018, Glynn et al., 2024, Lee et al., 2020, Jimbo et al., 1997, Shorstova et al., 2021). As for chemotherapy, no consensus exists on the indication of radiation therapy that was used in only 45 of 306 SCCOHT collected in a recent revie not allowing a conclusion to be drawn on its interest (Wens et al., 2023).

**Table 1:** Drugs used alone or in combination in patients with SCCOHT or in pre-clinical models.

Chemotherapy	
	Platinum (cisplatine)-etoposide
	Platinum (carboplatine)-paclitaxel
	Platinum-Bleomycin
	Platinum-Ifosfamide (Wang et al., 2017)
	Platinum-Doxorubicin

Immunotherapy	
	Anti-PD-L1: pembrolizumab, nivolumab (Blatnik et al., 2024, Jelinic et al., 2018)
	Anti-PD-L1 + anti-angiogenic + CDK4/6 inhibitor (cycline-dependent kinase): pembrolizumab + bevacizumab + abemaciclib (Glynn et al., 2024, Lee et al., 2020)
	Anti-CTLA-4 (Cytotoxic T-Lymphocyte Associated Protein 4)
	Multi-tyrosine kinase inhibitor: ponatinib, dasatinib (Lee et al., 2020)
	PARP Inhibitors: olaparib (Gao et al., 2024)
	Epigenetic Inhibitors (inhibition of the PRC2 catalytic subunit EZH2): tulmimetostat (Wang et al., 2017)
	Pan-HDAC inhibitors: quisinostat alone or in combination with EZH2 inhibitor
	BET (bromodomain and extra- terminal domain) family of proteins inhibitors: and Combinations of the BET inhibitor OTX015 targeting BET family member BRD2 (bromodomain containing 2) and MEK (mitogen-activated protein kinase kinase) inhibitors (Jimbo et al., 1997, Shorstova et al., 2021)

Two of the three patients underwent genetic testing, showing no pathogenic mutations. Longy et al. (Longy et al., 1996) reported familial SCCOHT, and Witkowski et al. demonstrated germline and somatic SMARCA4 mutations in about 95 % of cases, opening up new therapeutic options (Witkowski et al., 2014). Tischkowitz et al. noted that SMARCA4 inactivation increases sensitivity to CDK-4/6 inhibitors, while EZH2 overactivity may be targeted with specific inhibitors (Tischkowitz et al., 2020). SCCOHT tumors also express PD-L1, suggesting a potential benefit from immune checkpoint inhibitors (Table 1; 14, 25, 28–33). Despite these findings, the value of genetic testing for at-risk relatives remains debated, given the autosomal dominant inheritance. Surveillance for at-risk family members is challenging due to the lack of specific imaging findings, high risk of misdiagnosis, and uncertain benefit from risk-reducing surgery (Wade et al., 2025). Nonetheless, as Podwika et al. argue, genetic testing is crucial for informed decision-making in carriers of pathogenic variants. Risk-reducing surgery, such as bilateral salpingo–oophorectomy, may be considered in adolescents, with fertility preservation options (Podwika et al., 2020).

In conclusion, our case reports highlight the challenges of preoperative diagnosis for SCCOHT. Importantly, our data suggest that stage I SCCOHT is not always associated with a poor prognosis, provided that intensive multimodal treatment is administered.

Written informed consent was obtained from the patients for publication of this case report and accompanying images.

## CRediT authorship contribution statement

**J. Benichou:** Writing – original draft. **J. Varinot:** Data curation. **R. Bossi-Croci:** Data curation. **M. Bazot:** Data curation. **D. Sitbon:** Data curation. **M. Dahan:** Data curation. **C. Ferrier:** Data curation. **Y. Dabi:** Data curation. **C. Touboul:** Data curation. **J. Lotz:** Resources. **E. Darai:** Supervision, Data curation, Methodology.

## Declaration of competing interest

The authors declare that they have no known competing financial interests or personal relationships that could have appeared to influence the work reported in this paper.
