# Supplementary material for: Small cell carcinoma of the ovary hypercalcemic type (SCCOHT): About three case reports
Source: Gynecol Oncol Rep. 2025 Aug 26;61:101932. doi: 10.1016/j.gore.2025.101932 (PMC12419111; doi:10.1016/j.gore.2025.101932)
Supplement: Supplementary Data 1 [file mmc1.pdf]

Subscribe to DeepL Pro to translate larger documents.
Visit www.DeepL.com/pro for more information.

GHU PARIS-EST TENON Hospital

PATHOLOGICAL ANATOMY Department of Professor P. CALLARD

4, rue de la chine 75970 PARIS Cedex 20 ☎01 56 01 66 77 01 56 01 78 76

[redacted]

Gender: F

[redacted]

[redacted]

Prof. Jean-Pierre LOTZ
MEDICAL ONCOLOGY
TENON Hospital

75970 PARIS cedex 20

[redacted]

INTERNAL COPY 03/31/2025

TARCH

Clinical information:
Blocks submitted by Professor Jean-Pierre LOTZ for review of an ovarian tumor diagnosed as juvenile granulosa tumor, in a 22-year-old woman. Absence of hypercalcemia. Existence of estrogenic manifestations?

COMMUNICATION BLOCKS

2 blocks were sent to the laboratory, on which 2 HES slides + mucin staining and an immunohistochemical study using the peroxidase technique on kerosene sections were performed on the 1st block.

- BLOCK NO. P045279 IB:

* The slide shows a well-limited tumoral proliferation, slightly bumpy on the surface, bordered in one area by a very fine border of non-tumoral tissue that may correspond to ovarian parenchyma (3 mm), although no follicular structure is identified.
This tumor proliferation is very dense, very rich in cells. It is diffuse, consisting of patches of cells with no architecture, sometimes with very fine fibrous tracts lobulating it. Proliferation is artefactual, with tumour cells usually dissociated, making cytological analysis more difficult. However, the cells appear small or sometimes medium-sized, with sparse cytoplasm, large hyperchromatic or slightly vesicular nuclei, finely nucleolated and lacking a distinctive groove. Mitoses are very numerous. In some areas, particularly in the periphery, which may be better preserved, cells appear larger, with more abundant eosinophilic cytoplasm and large, finely nucleated, vesicular nuclei, always the site of multiple mitoses. Microcystized structures of various sizes can be seen, with empty lumens or filled with a pale, follicle-like eosinophilic substance. However, this substance does not stain like mucin. Endovascular tumor permeation is not detected.
* Reticulin staining is of moderate density, surrounding small islands and cell trabeculae.
* In the V immunohistochemical study using the peroxidase technique on kerosene sections, the tumor was completely negative for all cytokeratins tested (pancytokeratin AE1-AE3, CK 5/6, CK 7, CK 10/13, CK 19), with VEMA, Calretinin, hormone receptors, Beta HCG, lVAlpha-fetoprotein, lVHMB 45, chromogranin A and lVInhibin (verified 2 times with positive external control). Some scattered cells labelled with anti-Actin antibody and moderately labelled sectors with CD99 antibody are observed. Tumor proliferation is intensely and diffusely Vimentin-positive.

- BLOCK NO. P045279 IF:

Anteriorities: T05H00008, T05T00002, T04C02528, T04H08487Adicap: OHGO0185, OHGO0171, OHGOG7A0, OHGOX7E0, OHGOX7F0, OHGO0180, OHGO0150

This is a tumoral proliferation of similar appearance, with no healthy border. LV appearance is more artefactually dissociated.

CONCLUSION:

Difficult tumor to diagnose, presenting more morphological and immunohistochemical evidence for an undifferentiated small-cell carcinoma with large-cell sectors in its variety of hypercalcemic-type small-cell ovarian carcinoma, than for a tumor of the juvenile granulosa; to be compared with the clinic.

The slides were viewed in collaboration with DRS P.DUVILLARD and J.C. SABOURIN at the Institut G.ROUSSY where CK22, a 3rd Inhibin and Fli-1 will be applied.

PS: given the discrepancy with the clinic, a block was sent to Prof. YOUNG in BOSTON, USA.

Answered on 24/09/04 Doctor
Annie CORTEZ

REPORT ADDENDUM N°1

The additional application of anti-Desmine and Panleucocyte antibodies proved negative.

ADDITIONAL REPORT No. 2: reply from Professor Robert H. YOUNG of Massachusetts General Hospital, USA.

Professor Young confirms the diagnosis hypercalcemic small-cell ovarian carcinoma.
A photocopy of the reply is sent to Professor LOTZ at TENON Hospital.

14/10/2004, Dr Annie CORTEZ
